# Surgical Technique for Concurrent Endoscopic Carpal Tunnel Release and Distal Radius Fracture Fixation Using the Flexor Carpi Radialis Approach: A Case Series

**DOI:** 10.1016/j.jhsg.2021.11.007

**Published:** 2022-01-13

**Authors:** Abhiram R. Bhashyam, Dennis S. Kao

**Affiliations:** ∗ Department of Orthopaedic Surgery, Massachusetts General Hospital, Boston, MA; † Division of Plastic Surgery, University of Washington, Seattle, WA

**Keywords:** Carpal tunnel release, Distal radius fracture, Endoscopic, FCR approach

## Abstract

**Purpose:**

Multiple prior studies have assessed the results of open approaches for concurrent carpal tunnel release with distal radius fracture fixation; however, less is known regarding the feasibility of endoscopic techniques, especially in the setting of high-energy trauma. In this study, we assessed the feasibility and results of concurrent endoscopic carpal tunnel release and distal radius fracture fixation using the flexor carpi radialis approach after high- and low-energy trauma.

**Methods:**

We performed a retrospective, single-surgeon study of 17 consecutive adult patients (aged >18 years) who underwent open reduction internal fixation of an acute distal radius fracture with concurrent endoscopic carpal tunnel release at a level 1 trauma center between April 2017 and October 2020. Recovery from median nerve dysfunction was assessed from patient charts at routinely scheduled postoperative follow-up visits (at 2 weeks, 4 weeks, 6 weeks, and 12 weeks).

**Results:**

The transverse carpal ligament could be visualized and released in all patients. All patients had a return of light touch sensibility with or without intermittent paresthesia by 12 weeks after surgery (the median time from surgery to recovery was 19 days [range, 12–82 days]). There were no patient reports or clinical examination evidence of palmar cutaneous branch, recurrent motor branch, or the third common digital nerve injury. Time to recovery was significantly different in the setting of high- versus low-energy trauma (26 days vs 18 days, respectively; *P* = .02).

**Conclusions:**

In this study, we demonstrated that concurrent endoscopic carpal tunnel release using the flexor carpi radialis approach for distal radius fracture fixation in the settings of high- and low-energy trauma is safe from major complications and effective at releasing the transverse carpal ligament.

**Type of study/level of evidence:**

Therapeutic IV.

Distal radius fractures may trigger, reveal, or decompensate symptoms of median nerve dysfunction in 0.5% to 21% of cases.^1^^,^^2^ After a distal radius fracture, median nerve dysfunction can develop or worsen due to deformity or contusion secondary to fracture displacement, edema, or hematoma. In contrast to median nerve dysfunction that typically resolves after manipulative reduction, acute carpal tunnel syndrome progressively worsens after injury.^3^^,^^4^ In either setting, surgical fixation with median nerve decompression is indicated.^1^^,^^3^^,^^5^^,^^6^ In addition, although previous studies have shown no benefit to universal prophylactic neurolysis of the median nerve, intervention in higher-risk populations may still be indicated.^3^^,^^5^^,^^7^

Surgical release of the median nerve at the carpal tunnel is commonly performed using a wide volar, mini-open, or endoscopic technique.^1^^,^^6^ In the setting of a concurrent distal radius fracture, most surgeons avoid using a central longitudinal approach to the distal radius with the combined release of the transverse carpal ligament, given the unacceptable rate of postoperative median nerve symptoms.^8^ Three safer options for concomitant release of the median nerve at the carpal tunnel include the following: (1) a separate incision in the palm, (2) an extension of the flexor carpi radialis (FCR) incision across the wrist crease with identification of the palmar cutaneous branch of the median nerve, or (3) a separate endoscopic carpal tunnel release.^6^^,^^9^^,^^10^ Multiple prior studies have assessed the results of open approaches for concurrent carpal tunnel release; however, less is known regarding the feasibility and results of endoscopic techniques, especially in the setting of high-energy trauma.^1^^,^^7^^,^^9^, ^10^, ^11^, ^12^, ^13^, ^14^

In this study, we assessed whether an unconventional surgical technique for concurrent endoscopic carpal tunnel release and distal radius fracture fixation after high- and low-energy trauma resulted in improvement in median nerve light touch sensibility and whether there were associated complications. We hypothesized that high-energy trauma would be associated with a longer time to return of nerve sensory recovery.

## Materials and Methods

### Study design

This study was approved by University of Washington institutional review board. We performed a retrospective, single-surgeon study of 22 consecutive adult patients (aged >18 years) who underwent open reduction internal fixation of an acute distal radius fracture with concurrent endoscopic carpal tunnel release at an American College of Surgeons level 1 trauma center from April 2017 to October 2020. Patients were excluded if their fracture was treated with isolated dorsal bridge plating (4 patients). Of the 18 eligible patients, 17 patients completed the final evaluation of nerve recovery (94% follow-up rate) with a median follow-up of 9.7 weeks.

### Evaluation of carpal tunnel release, nerve function, and recovery

We assessed the ability of this technique to successfully release the transverse carpal ligament. We also measured the rate of conversion from endoscopic to open carpal tunnel release. The postoperative clinical follow-up notes were reviewed to determine the return of light touch sensibility, the prevalence of persistent or delayed median nerve dysfunction, early repeat surgery for carpal tunnel syndrome, and complications typically associated with the approach. Based on previous studies, median nerve dysfunction was defined as anesthesia, paresthesia, or dysesthesia referable to the thumb, index, middle, and/or radial half of the ring finger. Specific potential complications of the approach that were considered included palmar cutaneous branch injury (anesthesia, paresthesia, or dysesthesia referable to the thenar eminence), recurrent motor branch injury (inability to oppose the thumb), or injury to the third common digital nerve.^1^^,^^2^^,^^8^^,^^10^^,^^13^ Recovery from median nerve dysfunction was assessed from patient charts at routinely scheduled postoperative follow-up visits (at 2 weeks, 4 weeks, 6 weeks, and 12 weeks). Recovery was defined as the return of nerve sensibility to light touch (symmetric to the ipsilateral little finger and contralateral side) with or without intermittent paresthesia.^1^^,^^2^^,^^8^

### Independent variables

Detailed demographic and clinical data were identified for each patient using our institution’s electronic medical record (Table 1). Relevant variables including age, sex, injury mechanism, injury details (open fracture, concomitant injury, polytrauma), and pre-existing median nerve dysfunction were selected based on previous similar studies.^3^^,^^15^ Distal radius fractures were classified using the AO/Orthopaedic Trauma Association fracture classification by both authors.^16^ To mitigate interobserver variability during analysis, all fractures were subsequently grouped as extra-articular (23.A) or intra-articular (partial articular [23.B] and complete articular [23.C]). The energy of the injury mechanism was defined according to the Advanced Trauma Support guidelines. High-energy injuries included falls from a height (eg, ladder, roof, scaffolding 5+ stairs) or a motor vehicle collision. Patients who did not meet the criteria for high-energy trauma were considered low-energy trauma.^2^^,^^17^

**Table 1:** Demographic, Injury, and Treatment Characteristics of the Patient Cohort∗

Characteristics	All Responders	Low-Energy Trauma	High-Energy Trauma	*P* Value
	n = 17	n = 9	n = 8	
Demographic characteristics				
Age at trauma, y, median (range)	51 (26–76)	51 (29–71)	52 (26–76)	.70
Male (n, %)	5 (29%)	2 (22%)	3 (38%)	.62
Prior carpal tunnel symptoms (n, %)	5 (29%)	4 (44%)	1 (13%)	.29
Injury and treatment characteristics				
Dominant-side injury (n, %)	6 (35%)	5 (56%)	1 (13%)	.13
Mechanism of trauma (n, %)				…
Fall <3 m	9 (53%)	9 (100%)	0 (0%)	
Fall >3 m	4 (24%)	0 (0%)	4 (50%)	
Bicycle accident	2 (12%)	0 (0%)	2 (25%)	
Car accident	1 (6%)	0 (0%)	1 (13%)	
Motorcycle accident	1 (6%)	0 (0%)	1 (13%)	
Isolated injury (n, %)	10 (59%)	7 (78%)	3 (38%)	.15
Open fracture (n, %)	1 (6%)	0 (0%)	1 (13%)	.47
AO classification (n, %)				1.00
Type A	3 (18%)	2 (22%)	1 (13%)	
Type B	1 (6%)	1 (11%)	0 (0%)	
Type C	13 (76%)	6 (67%)	7 (88%)	
Intra-articular fracture (n, %)	14 (82%)	7 (78%)	7 (88%)	1.00
Use of dorsal spanning plate (n, %)	3 (18%)	1 (11%)	2 (25%)	.58
Outpatient procedure (n, %)	10 (59%)	7 (78%)	3 (38%)	.15

∗ *P* value–based Wilcoxon rank-sum or Fisher exact test comparing low-energy versus high-energy trauma.

### Surgical Technique

Patients were indicated for endoscopic carpal tunnel release on the basis of clinical diagnosis of persistent median nerve dysfunction despite adequate reduction and elevation.^1^^,^^10^^,^^13^^,^^15^ Median nerve dysfunction was diagnosed on the basis of the following: (1) acute or subacute onset of median nerve paresthesia, (2) numbness or sensory disturbance in the median nerve distribution of the injured extremity, or (3) the pre-existing carpal tunnel symptoms exacerbated by the injury.^2^^,^^5^

**Figure 1:**
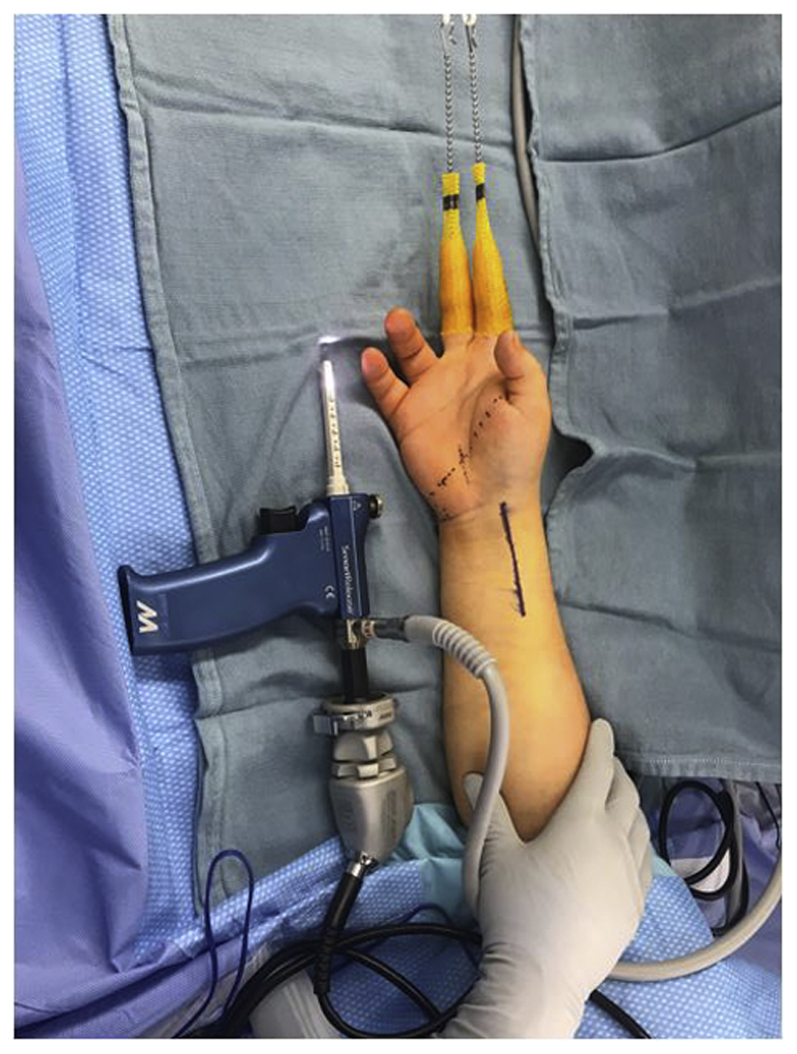
Demonstration of surgical setup, instrumentation, and skin markings for flexor carpi radialis approach.

**Figure 2:**
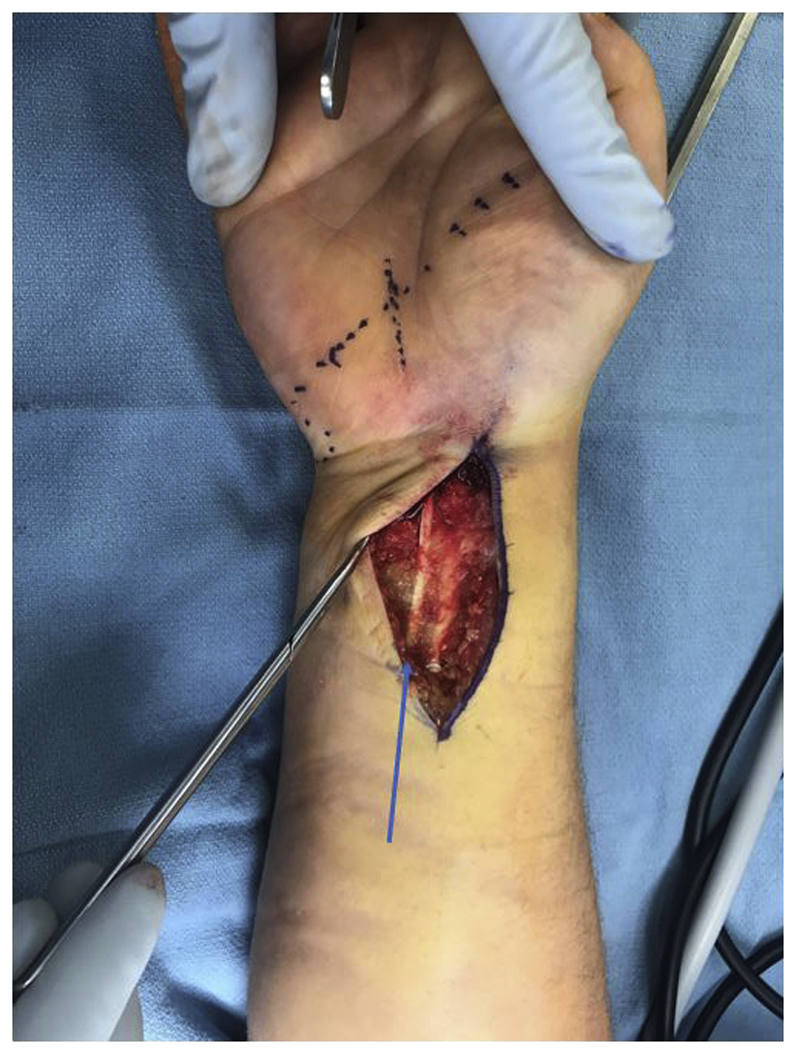
Demonstration of subcutaneous skin flap raised to the ulnar border of the palmaris longus (blue arrow).

All procedures under regional anesthesia were performed using a tourniquet (Video, available on the *Journal’*s website at www.jhsgo.org). Prior to incision, 4.5 kg of traction was applied using a pulley with a rope attached to the finger traps placed on the index and middle finger (Fig. 1). A provisional reduction was subsequently obtained using the Agee^18^ maneuver to reapproximate the normal bony and soft tissue anatomy at the wrist. Intraoperative fluoroscopy was used to confirm the appropriate reduction. A standard incision for the FCR approach was marked, and dissection was performed sharply down to the level of the FCR fascia. Dissection was performed bluntly toward the ulna in the subcutaneous plane above the level of the antebrachial fascia using loupe magnification (to avoid injury to the palmar cutaneous branch of the median nerve) until the ulnar border of the palmaris longus (PL) tendon was identified (Fig. 2). The PL was subsequently gently retracted radially using a Ragnell retractor to expose the antebrachial fascia underneath it. The antebrachial fascia ulnar to the PL was then incised longitudinally to gain access to the undersurface of the antebrachial fascia. Once this was achieved, the synovial elevator was introduced to bluntly peel off all soft tissue attachment on the undersurface of the antebrachial fascia and its distal extension (ie, undersurface of the transverse carpal ligament). If blood or a hematoma was encountered at this stage, it was irrigated and suctioned out using a Frazier tip suction to create a clean field for the endoscope passage. Endoscopic carpal tunnel release was subsequently performed as previously described by Agee et al^19^ by the sequential passage of dilators, followed by the camera-blade assembly to divide the transverse carpal ligament under endoscopic visualization. If adequate visualization could not be obtained, we planned to perform an open carpal tunnel release. Following carpal tunnel release, surgical fixation of the distal radius fracture can proceed in a typical fashion using the FCR approach.

### Statistical analysis

Baseline characteristics and clinical results between patients with high- and low-energy trauma were compared using Fisher exact test for categorical variables and *t* test for continuous variables.^3^^,^^15^ *P* values of <.05 were considered statistically significant.

## Results

### Characteristics of patient population

In this cohort of 17 patients, 12 patients were women (71%), and the median age was 51 years (range, 26–76 years). Five patients had initial symptoms of carpal tunnel syndrome. Among all injuries, 6 occurred on the dominant side (35%) and 8 were the result of high-energy trauma (47%). Ten fractures (59%) were isolated injuries, 14 were intra-articular (82%), and only 1 was open. The median time from injury to surgery was 6 days (range, 0–19 days). Ten patients were treated as an outpatient (59%); fragment-specific fixation was used in 2 patients (volar locked plating for the remainder of patients), and a supplemental dorsal spanning plate was used to augment fixation in 3 patients. In this cohort, patients with low- versus high-energy trauma were similar in their demographic, injury, and treatment characteristics (Table 1).

### Evaluation of carpal tunnel release, nerve function, and recovery

The transverse carpal ligament was released completely endoscopically in all patients without conversion to an open procedure. No patients required revision carpal tunnel release in our follow-up period.

The median follow-up duration for the evaluation of nerve function was 9.7 weeks (range, 1.7–102 weeks). Among all 17 patients, 15 patients (88%) demonstrated a return of median nerve light touch sensibility by 12 weeks after surgery. Improvement of nerve sensibility with or without intermittent paresthesia was observed in all 17 patients. Two patients described infrequent, intermittent paresthesia at the tip of the thumb and index finger. There were no patient reports or clinical examination evidence of palmar cutaneous branch, recurrent motor branch, or the third common digital nerve injury. The likelihood of nerve recovery was similar in the setting of high- or low-energy trauma (Table 2).

**Table 2:** Results of Patient Cohort Subdivided by Low-Energy Versus High-Energy Trauma∗

Results	All Responders	Low-Energy Trauma	High-Energy Trauma	*P* Value
	n = 17	n = 9	n = 8	
Time from surgery to the final follow-up, wk, median (range)	9.7 (1.7–101.6)	8.9 (1.7–101.6)	10.7 (2.7–42.7)	
Resolution of median nerve dysfunction with return of light touch sensibility (n, %)	15 (88%)	9 (100%)	6 (75%)	.21
Return of light touch sensibility +/− intermittent paresthesia (n, %)	17 (100%)	9 (100%)	8 (100%)	
Time from surgery to return of light touch sensibility +/− intermittent paresthesia, d, median (range)	19 (12–82)	18 (12–40)	26 (19–82)	.02
Total patients at 2 weeks after surgery (n, % of subgroup)	4 (24%)	4 (44%)	0 (0%)	
Total patients at 4 weeks after surgery (n, % of subgroup)	12 (71%)	7 (78%)	5 (63%)	
Total patients at 6 weeks after surgery (n, % of subgroup)	14 (82%)	9 (100%)	5 (63%)	
Total patients at 12 weeks after surgery (n, % of subgroup)	17 (100%)	9 (100%)	8 (100%)	

∗ *P* value–based Wilcoxon rank-sum or Fisher exact test comparing low-energy versus high-energy trauma.

**Figure 3:**
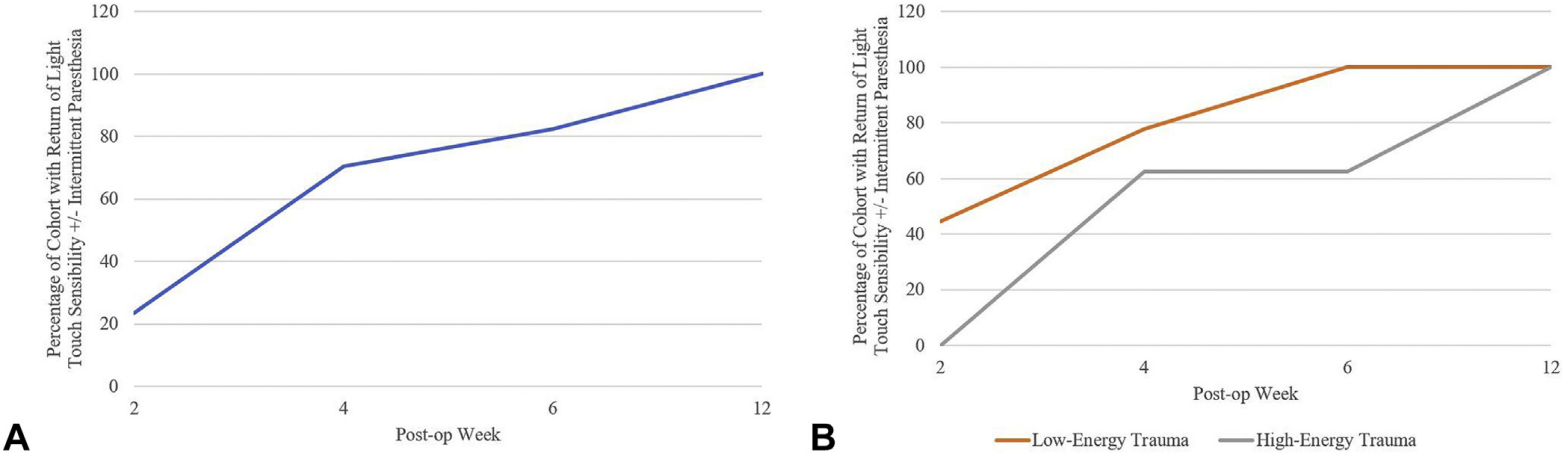
A Percentage of the full cohort with the return of light touch sensibility +/− intermittent paresthesia. B Delay in time to recovery between high- and low-energy trauma. Video. Clinical video illustrating the surgical approach.

The median time from surgery to return of light touch sensibility with or without intermittent paresthesia was 19 days (range, 12–82 days) for the entire cohort (Fig. 3A). Time to recovery was significantly different in the setting of high- versus low-energy trauma (26 days vs 18 days, respectively; *P* = .02). In the low-energy trauma subset, 4 patients recovered by 2 weeks after surgery, and all patients had recovered by 6 weeks after surgery. In contrast, in the high-energy trauma subset, none of the patients in the high-energy group had recovered by 2 weeks after surgery, and 3 patients continued to recover between 6 and 12 weeks after surgery (Table 2, Fig. 3B).

## Discussion

In this study, we demonstrated the feasibility of a technique for concurrent endoscopic carpal tunnel release and distal radius fracture fixation using the FCR approach that allows for the following: (1) avoids the glabrous skin of the proximal palm, (2) provides adequate exposure of the distal radius, (3) reliably decompresses the median nerve, (4) releases the distal antebrachial fascia, and (5) avoids injury or excessive manipulation of critical neurologic structures.^7^ The transverse carpal ligament was successfully released endoscopically in all patients without conversion to an open approach. There were no patient reports or clinical examination evidence of palmar cutaneous branch, recurrent motor branch, or the third common digital nerve injury. Light touch sensibility with or without intermittent paresthesia in the median nerve distribution after the release of the transverse carpal ligament improved in all patients by 12 weeks after surgery.

Previous studies have described the feasibility and results of other carpal tunnel release techniques in the setting of distal radius fractures. Multiple variations of open release have been described. The simplest technique is to make a separate approach in the palm, accepting the need for the second incision and the potential for pillar pain.^7^ Other open techniques rely on modifications of the approach used for distal radius fixation. Lattmann et al^8^ reported on the use of a single-incision midline volar approach; however, this had an unacceptably high rate of persistent median nerve symptoms.^12^ Instead, Orbay et al^11^ recommended using an extended volar approach beyond the wrist crease while avoiding injury to the palmar cutaneous branch of the median nerve. Extending the approach by Orbay et al,^11^ Gaspar et al^12^ advocated an extensive approach to the carpal tunnel by dissecting all important structures crossing its path (including the recurrent thenar branch, palmar cutaneous branch, and the radial artery and its branches). A major criticism of this approach has been its technical difficulty requiring meticulous dissection and a longer incision.^12^ A simpler approach was proposed by Pensy et al^7^ as an extension of an earlier work by Weber and Sanders.^14^ They proposed a volar extensile approach by extending Henry’s approach to the carpal tunnel through the FCR sheath to avoid making 2 adjacent approaches.^7^^,^^10^ The primary disadvantages of this technique are the inability to directly visualize the distal portion of the release of the transverse carpal ligament (tested indirectly by the smooth passage of the surgeon’s little finger) and poor visualization of the recurrent motor branch on the radial aspect of the release.^7^^,^^10^^,^^14^

In contrast to open techniques, the feasibility of endoscopic approaches to carpal tunnel release concurrently with distal radius fixation is more limited. Endoscopic techniques are attractive given that the previous studies have demonstrated no significant difference in outcomes between open and endoscopic approaches; endoscopic techniques provide visualization of the entire transverse carpal ligament without requiring the second incision in the palm, and minimally invasive/endoscopic approaches are increasingly desired by patients.^19^^,^^20^ Badia et al^21^ was the first to propose an endoscopic technique; however, their approach used 2 distinct incisions (an extensive FCR approach for fracture fixation and a separate small transverse approach for endoscopic neurolysis). Low and Cheah^9^ published a technical note on the possibility of endoscopic carpal tunnel release using the Henry approach; however, the feasibility and results in a clinical setting were not assessed. Zemirline et al^1^ were the first to present a clinical case series in 10 patients. However, in their approach, all cases were in the setting of low-energy trauma treated in an outpatient setting with an unclear duration of recovery. In addition, their approach used an interval between FCR and PL, creating a potential for injury to the recurrent motor branch distally, and 5 of the 10 patients had complications related to persistent median/palmar cutaneous branch paresthesia or chronic regional pain syndrome.

Based on this literature review, current techniques of concurrent median nerve decompression by open or endoscopic techniques at the time of distal radius fracture fixation may increase overall morbidity and add unnecessary time and cost.^7^ In particular, commonly cited disadvantages to current techniques include the following: (1) pillar pain may persist for several months after incisions in the proximal palm, (2) the median nerve and its branches may be at risk during release or affected by adjacent scar with exposures overlying its course, and (3) endoscopic techniques may be more technically challenging in the setting of an unstable or displaced distal radius fracture with soft tissue injury and hematoma obscuring normal tissue planes.^6^^,^^7^ Our endoscopic approach addresses each of these limitations: (1) it uses the same FCR incision that is the most common exposure for volar plating, (2) it uses a safe interval on the ulnar aspect of PL to avoid potential injury to the recurrent motor branch or palmar cutaneous branch of the median nerve as described by Agee et al,^19^ and (3) the placement of traction helps to obtain a provisional reduction and restore near-normal soft tissue anatomy to make the endoscopic approach technically easier. With respect to cost, recent studies have demonstrated that open and endoscopic carpal tunnel release is equally cost effective if major nerve injury is avoided.^22^, ^23^, ^24^ In this series of 17 concurrent patients, we did not have to convert to an open release and no complications were observed, thereby demonstrating the technique’s feasibility. Key technical tips with this approach are as follows: (1) using traction to obtain a provisional reduction, (2) raising a thick skin flap until the ulnar border of PL is identified, and (3) using the synovial elevator to remove any blood or hematoma to ensure clear visualization of the transverse carpal ligament.

Previous studies have proposed that the energy transmission during trauma may influence the likelihood of developing median nerve dysfunction.^3^^,^^6^^,^^15^ A major concern for endoscopic approaches in settings of high-energy trauma is technical difficulty in safe visualization due to disruption of normal tissue planes secondary to fracture displacement and hematoma.^7^ Therefore, we specifically demonstrated the feasibility of our approach in the settings of high- and low-energy trauma. In addition, previous studies have investigated the energy associated with trauma as a risk factor for the development of median nerve dysfunction but did not distinguish between results or on the time course of recovery related to high-energy injury mechanisms.^2^^,^^3^^,^^15^ Chauhan et al^25^ demonstrated no statistical or clinically significant differences in patient-reported outcomes after distal radius fracture fixation with or without carpal tunnel release but did not differentiate results based on injury mechanism. Similarly, we found that nerve function 12 weeks after the surgery was comparable between high- and low-energy trauma groups using our endoscopic technique, although nerve function in the setting of high-energy injury mechanism took longer to recover. In addition, in the high-energy trauma group, some patients have persistent median nerve symptoms (ie, intermittent paresthesia) 10 months after surgery.

### Limitations

This study has several limitations. There are several potential biases inherent to a retrospective, single-surgeon study design. However, this study was designed to be limited in scope to address the feasibility and results of the described technique. Our cohort was limited to 17 patients, and not all potential predictors of nerve recovery could be assessed (although, to our knowledge, our cohort size is the largest for any report on the use of a concurrent endoscopic carpal tunnel release technique with distal radius fracture fixation). Based on this work, future retrospective and prospective studies in larger patient groups would be able to compare the results of open versus endoscopic techniques in high- and low-energy injury mechanisms. Although the duration of clinical follow-up was adequate to achieve our study objectives, further investigation with longer-term follow-up may provide additional insight. Finally, formal patient-derived outcome measures were not used to assess the qualitative results of the treatment. In addition, given the retrospective nature of this study, limited outcome measures were assessed (patient-reported description of sensation, light touch sensibility, and injury to a palmar cutaneous branch of median nerve/recurrent motor branch/the third common digital nerve). Future studies using validated outcome measures and more objective measures (such as standardized 2-point discrimination and monofilament testing) may add valuable information regarding treatment for this cohort of patients.
